# On the border of the amyloidogenic sequences: prefix analysis of the parallel beta sheets in the PDB_Amyloid collection

**DOI:** 10.1515/jib-2020-0043

**Published:** 2021-07-26

**Authors:** Kristóf Takács, Vince Grolmusz

**Affiliations:** PIT Bioinformatics Group, Eötvös University, Budapest H-1117, Hungary; Uratim Ltd., Budapest H-1118, Hungary

**Keywords:** amyloid, amyloid-precursor, amyloidogenic proteins, PDB, prefix, suffix

## Abstract

The Protein Data Bank (PDB) today contains more than 174,000 entries with the 3-dimensional structures of biological macromolecules. Using the rich resources of this repository, it is possible identifying subsets with specific, interesting properties for different applications. Our research group prepared an automatically updated list of amyloid- and probably amyloidogenic molecules, the PDB_Amyloid collection, which is freely available at the address http://pitgroup.org/amyloid. This resource applies exclusively the geometric properties of the steric structures for identifying amyloids. In the present contribution, we analyze the starting (i.e., prefix) subsequences of the characteristic, parallel beta-sheets of the structures in the PDB_Amyloid collection, and identify further appearances of these length-5 prefix subsequences in the whole PDB data set. We have identified this way numerous proteins, whose normal or irregular functions involve amyloid formation, structural misfolding, or anti-coagulant properties, simply by containing these prefixes: including the T-cell receptor (TCR), bound with the major histocompatibility complexes MHC-1 and MHC-2; the p53 tumor suppressor protein; a mycobacterial RNA polymerase transcription initialization complex; the human bridging integrator protein BIN-1; and the tick anti-coagulant peptide TAP.

## 1 Introduction

Amyloids are misfolded protein aggregates, which are present in numerous biological organisms as structural building blocks or immunological agents [1–7]. In humans, the amyloid formation is frequently associated with neurodegenerative diseases and abnormal metabolic conditions [8–11].

The structural studies of amyloid aggregates were considered to be difficult until recently, since being aggregates, they cannot be crystallized and measured by X-ray diffractometry. With the recent developments of solid-state NMR and cryo-electron microscopy, dozens of amyloid structures were deposited in the Protein Data Bank (PDB) [12, 13] in the past several years.

With the more than 100 amyloid structures among the PDB’s 174 thousand entries, it is now possible to define structural characteristics, which well-describe amyloid structures. One good approach was made by [13], where the authors, with the application of a combination of textual search and specific geometric conditions, successfully retrieved the known amyloid structures from the PDB.

In a recent work of ours [14], we have defined a geometric set of constraints, by which we selected all the amyloid molecules, found by the method of [13], plus numerous globular proteins, with partial amyloid-like substructures. We emphasize that we were using geometric constraints for the β-sheet regions in the coordinate sections of the PDB files, without *any* textual search in the annotation section of those files. Since the annotation sections of PDB files are known to be less reliable than the coordinate sections, this technique increases the reliability of our results, and, additionally, helps in devising the proper definition of the amyloid-like structures. The resulting selection of the PDB entries, called the PDB_Amyloid list, is available as an automatically and regularly updated list of PDB entries, at the site http://pitgroup.org/amyloid/. Since, on the average, around 30 new PDB entries are deposited every day, the “automatic update” feature is clearly necessary for this service. The PDB_Amyloid list contains more than 660 entries today.

The geometric constraints, applied in [14], are as follows:

(i) First, parallel β-sheet segments are identified. The parallel segments need to be on separate polypeptide chains, their distance needs to be between 2 and 15 Å, and the standard deviation of their distance needs to be less than 1.5 Å.
(ii) Second, the large curvature parallel segments are excluded;
(iii) Third, the parallel segments need to cover at least the one-seventh of the length of the whole chain.

For a more detailed mathematical description of the constraints above, we refer to the original article [14].

We note that the requirement of considering only segments on separate polypeptide chains efficiently excludes hairpin and β-barrel structures, and also partial molecular structures, labeled as “amyloids”, but lacking the repeated, parallel β-sheets in the PDB-deposited files.

### 1.1 Prefixes

The more than 660 PDB entries, available at http://pitgroup.org/amyloid/, yield a rich set of amyloid-related molecular structures. The list of the globular proteins (i.e., not the misfolded, aggregated amyloid structures in the list) have a special feature: these molecules remained soluble, but they have partial sub-structures, satisfying the conditions (i), (ii) and (iii) above.

Therefore, the globular, soluble proteins from these 660 entries are “suspiciously amyloidogenic” since they satisfy the strict geometric constraints characteristic to amyloids but they are not amyloid structures. We assume that some mechanisms regulate or modulate their transition into amyloid state. The most straightforward assumption is that the borders of the beta-sheets contain short amino acid sequences, which can modulate the amyloid state of these molecules. Therefore, we intended to collect the short prefixes of these beta sheets, and study their appearances in other proteins in the Protein Data Bank.

In the present contribution, we consider the prefixes of the parallel *β*-sheets of the entries of the PDB_Amyloid list http://pitgroup.org/amyloid/. These prefixes are the starting subsections, where the order of the residues is the default N-terminus through C-terminus.

Here we identify the most frequently found prefixes in the PDB_Amyloid list, and then we search for them in the *complete* Protein Data Bank.

Our research hypothesis is that these prefixes, on the border of β-sheets, satisfying the properties (i), (ii) and (iii), have particular roles in structural changes to amyloid states under certain circumstances.

We identify numerous interesting hits, which have proven connections to amyloid formation. We stress that the hits, analyzed below, are found by sequence-searches in the whole PDB, without using *any* additional structural constraints, where the sequences we searched for were the prefixes, identified in the PDB_Amyloid list.

## 2 Methods

First, the defining parallel β-sheets, satisfying the conditions (i), (ii) and (iii) in the Introduction, of the PDB_Amyloid list http://pitgroup.org/amyloid/, were identified.

Next, we collected the length-5 prefixes of the form XXYYY, where the first three residues of the parallel *β*-sheet are YYY, and the last two residues, preceding the parallel section of the *β*-sheet, are XX.

**Figure 1:**
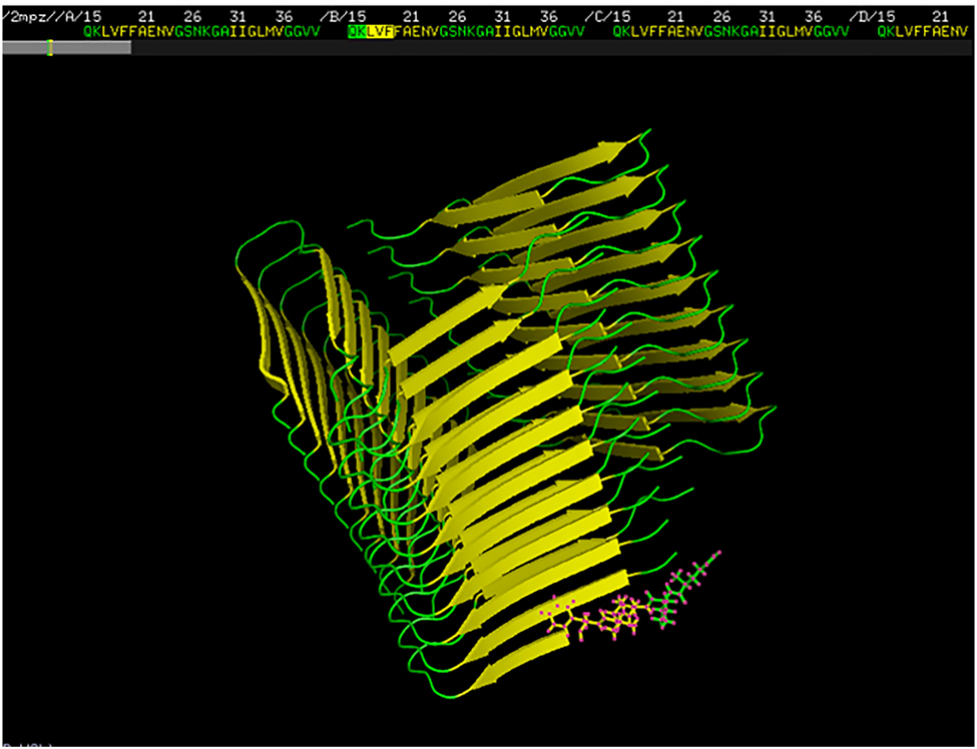
PDB entry 2MPZ, depicted with PyMol. Yellow color denotes β-sheets. The QKLVF prefix, where QK is green (it is not a part of the parallel β-sheet) and LVF is yellow (i.e., LVF are the first three residues of the parallel β-segment), is emphasized at the right bottom of the figure, while its corresponding sequence at the top center.

As an example for assigning XX and YYY parts of the prefix, we refer to Figure 1: it depicts the GLN-LYS-LEU-VAL-PHE (QKLVF) prefix from the amyloid structure of the PDB entry 2MPZ. Here XX corresponds to GLN-LYS, and YYY to LEU-VAL-PHE: GLN and LYS are not part of the *β*-sheet (they are colored by green), the first three residues are LEU, VAL and PHE (colored by yellow). Therefore, the XXYYY five-tuple catches the starting triplet (YYY in general, or LVF in the example of Figure 1) of the *β*-sheet, with the preceding two, non-*β*-sheet residue (XX in general, or QK in Figure 1). Therefore, the prefix describes the boundary of the β-sheet, with two residues just preceding the β-sheet, and the first three residues of the β-sheet.

Next, we have counted the number of appearances of the prefixes in the parallel *β*-sheet segments in the PDB_Amyloid list http://pitgroup.org/amyloid/. Note that one PDB entry may contain more than one identical prefixes (like in the case of PDB entry 2MPZ, shown in Figure 1). Therefore, the prefix counts contain multiplicities of two types: (i) multiple appearances in the very same PDB entry, or (ii) multiple appearances in different – and possibly homologous – PDB entries. Instead of introducing an unnecessarily complex homogeneity-corrected counting method for the prefix- and suffix appearances in the PDB_Amyloid list, we just count their raw, uncorrected number of appearances. Since the inclusion or exclusion of the protein structures in the PDB mostly relate to the interest of researchers depositing the structures, and do not carry a statistical or biological meaning. Moreover, we do not count the appearances in the whole PDB, just in the amyloid-like sublist of PDB_Amyloid. These counts (either corrected or uncorrected) can only be used informally, and do not show the frequency of these subsequences in the protein structures in Nature.

## 3 Discussion and results

In what follows, we consider the PDB_Amyloid list and note if the prefix appears in structures, described by the application of NMR spectroscopy (both solid and liquid phase), and X-ray crystallography.

Table 1 lists the first four, most frequently appearing prefixes in the NMR-identified structures from PDB_Amyloid, plus GGERA, the most frequent prefix in the X-ray crystallography identified structures in PDB_Amyloid.

**Table 1:** The four most frequent prefixes in the *β*-sheets of the NMR-identified entries of the PDB_Amyloid list, plus the most frequent prefix (GGERA) in the X-ray crystallography identified entries of PDB_Amyloid.

Prefix	Count	Appearance in the PDB_Amyloid list
QKLVF	77	2LMN, 2LMO, 2LMP, 2LMQ, 2LNQ, 2MPZ
GEYFT	48	1OLG, 1SAE, 1SAF, 1SAK, 1SAL, 3SAK
HHQKL	36	2LMO, 5KK3
HQKLV	25	2LMN, 2LMO, 2LMP, 2LMQ
GGERA	106	1DW9, 1DWK, 2IU7, 2IV1, 2IVQ, 2Y42

The second column gives the count of the appearances of the prefixes (counting the multiplicities) in the entries, listed in the third column.

From Table 1, the third most frequent prefix, HHQKL was not associated with amyloid-related PDB hits. The others are reviewed below.

### 3.1 The QKLVF prefix

The QKLVF prefix (i.e., GLN-LYS-LEU-VAL-PHE) appears 77 times in the NMR-identified members of the PDB_Amyloid list, in the following PDB-structures: 2LMN, 2LMO, 2LMP, 2LMQ, 2LNQ, 2MPZ. The 2MPZ structure is depicted in Figure 1. We are interested in the appearances of the QKLVF subsequence in the whole PDB, and we intend to identify the protein structures, which have the proven potential to turn to amyloids.

Among the numerous β-amyloid hits, which are not reviewed here, several interesting appearances of the QKLVF subsequence in globular proteins are in the T-cell receptors (TCR), bound with the major histocompatibility complexes MHC-1 and MHC-2, in the PDB entries 4P5T, 4OZF, 3QIU, 3QIW, 1BD2, 2IAN, 2IAM, 2IAL, 4WW1, 4WW2, 5XOT. Very interestingly, the misfolded MHC molecules in activated T-cells have a signaling function [15]. Additionally, the MHC molecule is known to misfold in several cases when in complex with TCR, and then it is degraded by housekeeping enzymes [16]. These articles show that the normally globular MHC molecules are known to misfold if in complex with the TCR molecule, containing the QKLVF subsequence.

### 3.2 The GEYFT prefix

**Figure 2:**
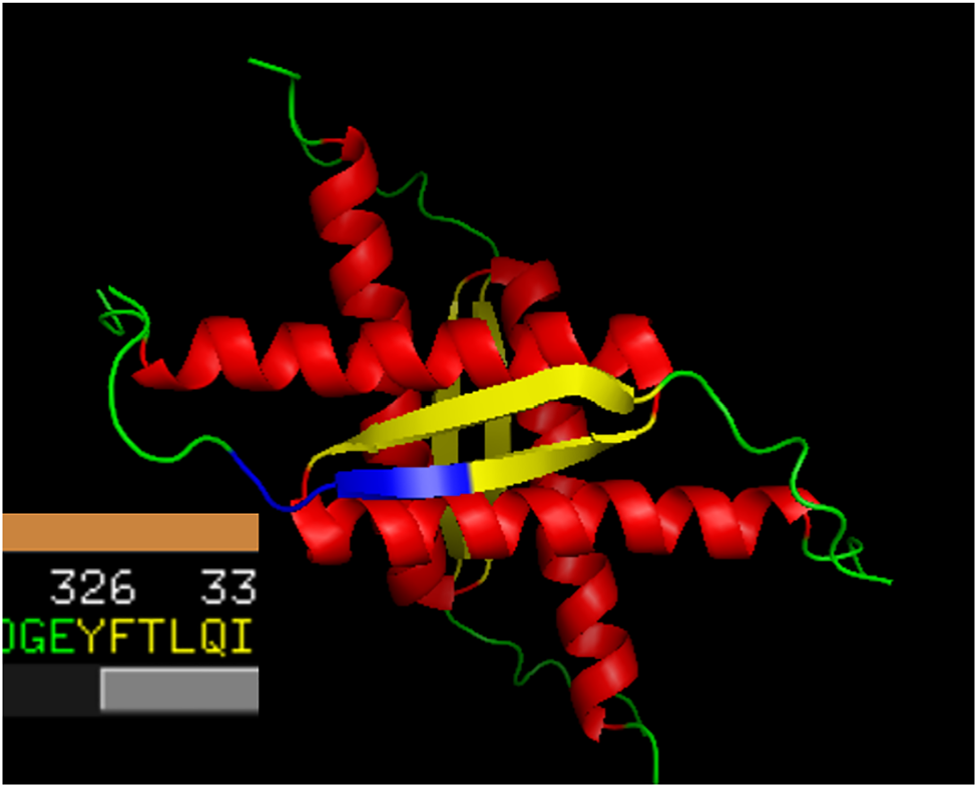
PDB entry 1OLG, depicted with PyMol. Yellow color denotes β-sheets. The GEYFT prefix, where GE are green (it is not a part of the parallel β-sheet) and YFT are yellow (i.e., YFT are the first three residues of the parallel β-segment), is emphasized at the left middle section of the figure, while its corresponding sequence at the lower left corner.

The GEYFT prefix (GLY-GLU-TYR-PHE-THR) appears 48 times in the NMR-identified members of the PDB_Amyloid list, in the PDB entries 1OLG, 1SAE, 1SAF, 1SAK, 1SAL, 3SAK. These are non-amyloid structures. One of them, 1OLG is depicted in Figure 2.

Numerous GEYFT appearances in the PDB_Amyloid list and also in the whole PDB are in p53 structures. p53 is a major tumor suppressor protein, whose gene is mutated in half of the human cancers [17–19], and both its mutational deficiency in humans and the knock-out of its gene in mice imply early on-set cancers [20, 21]. It is very surprising that p53 mutations have a tendency of prion-like, contagious amyloid transitions: it is found that the amyloid-like aggregation plays a role in the loss of the p53 function in several organisms and cell types [22, 23].

We note that identifying non-amyloid p53 structures in the PDB_Amyloid list shows the power of the methods by which the PDB_Amyloid list was created [14]: p53, an important non-amyloid structure with amyloidogenic properties is found in the list. We also note that numerous appearances of the GEYFT sequence in the whole PDB are also found in the p53 proteins.

Many GEYFT prefixes in the whole PDB are found in *Mycobacterium* (either *tuberculosis* or *smegmatis*) RNA polymerase transcription initialization complexes (e.g., 6DVC, 6JCX, 6JCY, 5ZX2). While it is not documented that these initialization complexes form amyloids, other bacterial transcriptional regulators do form amyloids. The *Bacillus subtilis* HeID, an RNA polymerase interacting helicase forms amyloids, as it was reported recently in [24]. Another finding that a mycobacterial global transcriptional factor, CarD, also forms amyloids, both *in vivo* and *in vitro* [25]. Therefore, it would not be surprising if the GEYFT-containing mycobacterial RNA polymerase transcription initialization complexes also formed amyloid fibrils.

### 3.3 The HQKLV prefix

**Figure 3:**
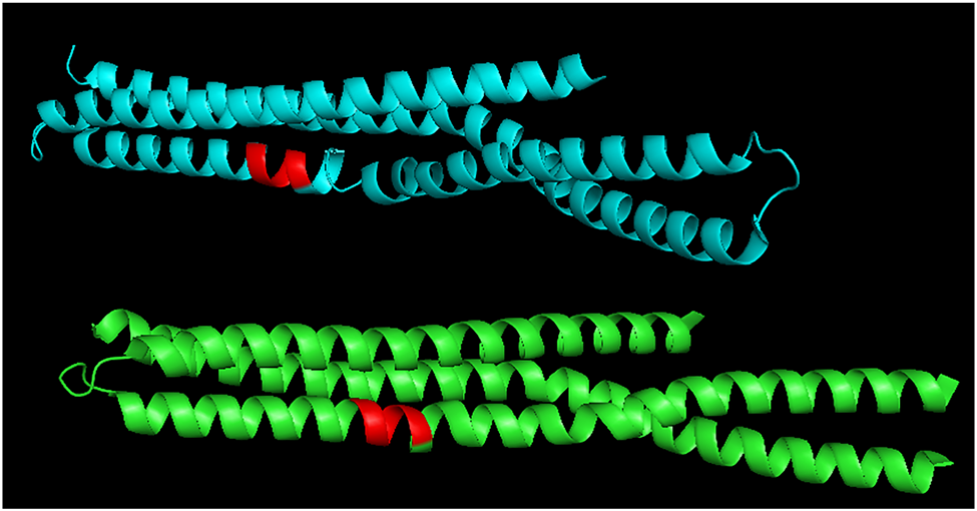
PDB entry 2FIC: the BAR domain of the human Bin1/amphiphysin II, depicted with PyMol. The red colored sections of the α-helices correspond to the HQKLV subsequence.

The HQKLV prefix (HIS-GLN-LYS-LEU-VAL) appears 25 times in the NMR-identified members of the PDB_Amyloid list, in the PDB entries 2LMN, 2LMO, 2LMP, 2LMQ; these are all β-amyloid fibrils. If we search for the HQKLV subsequence in the whole PDB, we find numerous amyloid structures and some human amphiphysins: human amphiphysin isoform 1 (PDB codes 3SOG, 4ATM), and human BIN1/amphiphysin II (2FIC). Interestingly, the HQKLV subsequence appears in these pure α-helix BIN1-structures as the part of the helix (Figure 3).

BIN1 is not known to form amyloid-fibrils, but it is well-known to relate to late-onset alzheimer’s-disease: its gene is the second most important risk locus for Alzheimer’s disease (after APOE: apolipoprotein E) [26], it is related to increased susceptibility for Alzheimer’s disease [27]. More recently, it was shown that BIN1 regulates BACE trafficking and β-amyloid production. Therefore, we may conjecture that the HQKLV subsequence plays a role in amyloid-formation, even if it is in an α-helix in BIN1 structures (Figure 3).

### 3.4 The GGERA prefix

**Figure 4:**
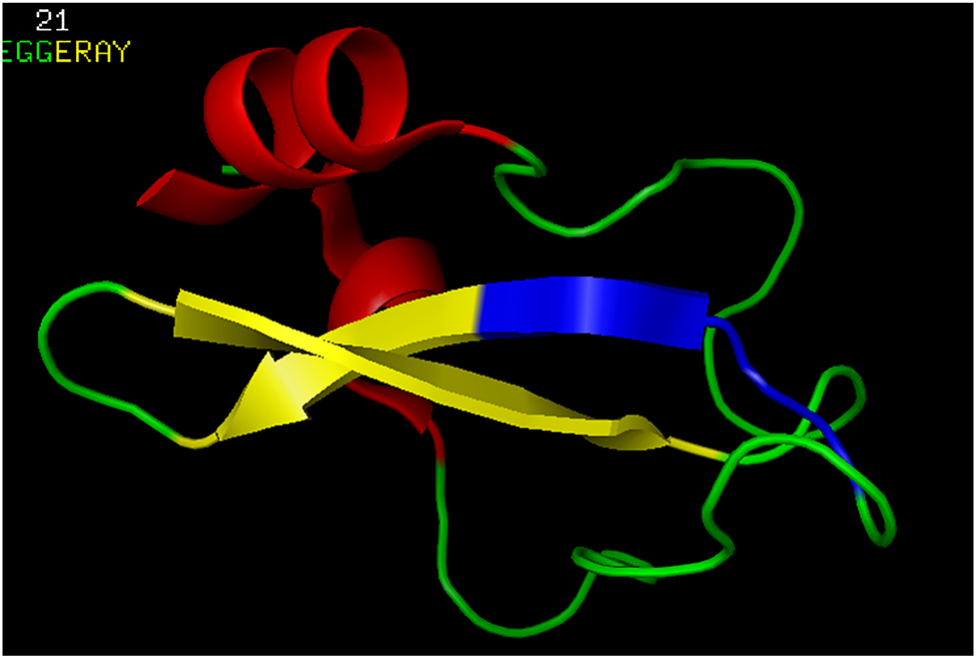
PDB entry 1TCP: the tick anticoagulant peptide (TAP), depicted with PyMol. The blue-colored section correspond to the GGERA prefix: GG preceeding the first three residues, ERA, in the β-sheet.

The GGERA prefix (GLY-GLY-GLU-ARG-ALA) appears 106 times in the X-ray crystallography-identified members of the PDB_Amyloid list, in the PDB entries 1DW9, 1DWK, 2IU7, 2IV1, 2IVQ, 4Y42; these are all bacterial cyanases. If we search for the GGERA subsequence in the whole PDB, the most interesting hit is the structure 1TCP: this is a tick anticoagulant peptide (TAP). The position of the GGERA subsequence is depicted in Figure 4.

The β-sheet prefixes, listed above, were all related to prion- or amyloid-formation. Here, GGERA is found in an anti-coagulant molecule: the tick anti-coagulant peptide. Therefore, the GGERA subsequence is

– the prefix of the parallel β-sheet sections of several soluble proteins (cyanases) from the PDB_Amyloid list, therefore the β-sheet, which starts with the GGERA sequence, is similar to those in the amyloid-structures, by satisfying properties (i), (ii) and (iii), listed in the Introduction;
– but the cyanases 1DW9, 1DWK, 2IU7, 2IV1, 2IVQ, 4Y42 are all soluble proteins.

Consequently, since GGERA also appears in the anti-coagulant 1TCP, it may have anti-amyloidogenic properties.

## 4 Conclusions

We have considered the PDB_Amyloid list of more than 660 PDB entries, identified by strict geometric rules, characterizing the known amyloid structures. Next, the length-5 prefixes of the β-sheet segments of these structures at http://pitgroup.org/amyloid/ were identified, and the most frequently appearing prefixes (i.e., QKLVF, GEYFT, HQKLV, and GGERA) were searched for in the whole PDB dataset. Surprisingly, simply by looking for these length-5 sequences, we have found such very interesting hits (besides the well-known amyloid structures) in the PDB like the

– T-cell receptor TCR, bound with the MHC-1 and MHC-2 major histocompatibility complexes;
– Major tumor suppression protein p53;
– Mycobacterium RNA polymerase transcription initialization complexes;
– BIN1/amphiphysin II structures;
– Tick anti-coagulant peptide (TAP);

which are either directly or indirectly connected to drastic structural changes (prion or amyloid formation, or anti-coagulant properties). Therefore, the prefixes of β-sheets, satisfying amyloid-like geometric properties in globular proteins may have characteristic roles in moderating amyloid-like structural transformations.

## Data availability

The automatically updated PDB_Amyloid web page is available at http://pitgroup.org/amyloid/. The list of the PDB codes of the PDB_Amyloid can be viewed and downloaded at http://pitgroup.org/apps/amyloid/amyloid_list.

## Supplementary Material

Supplementary Material Details

Supplementary Material Details

Supplementary Material Details

Supplementary Material Details
