# Supplementary figures and images for: On the border of the amyloidogenic sequences: prefix analysis of the parallel beta sheets in the PDB_Amyloid collection
Source: J Integr Bioinform. 2021 Jul 26;19(1):20200043. doi: 10.1515/jib-2020-0043 (PMC9069647; doi:10.1515/jib-2020-0043)

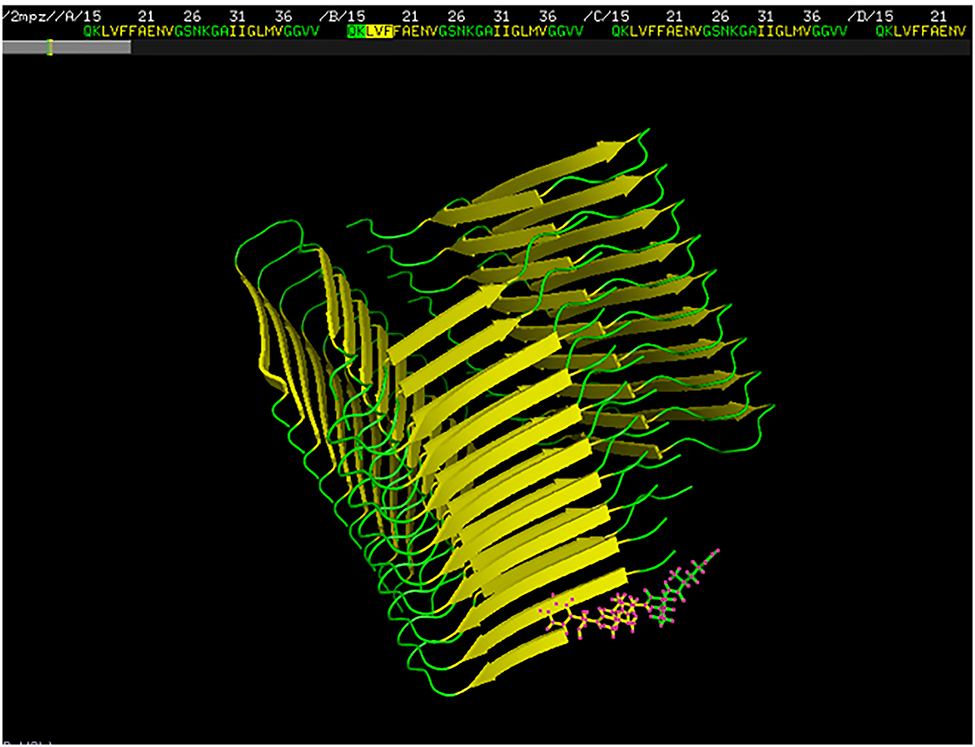

Supplement: Supplementary file 1 — Supplementary Material Details [file j_jib-2020-0043_suppl_001.jpg]

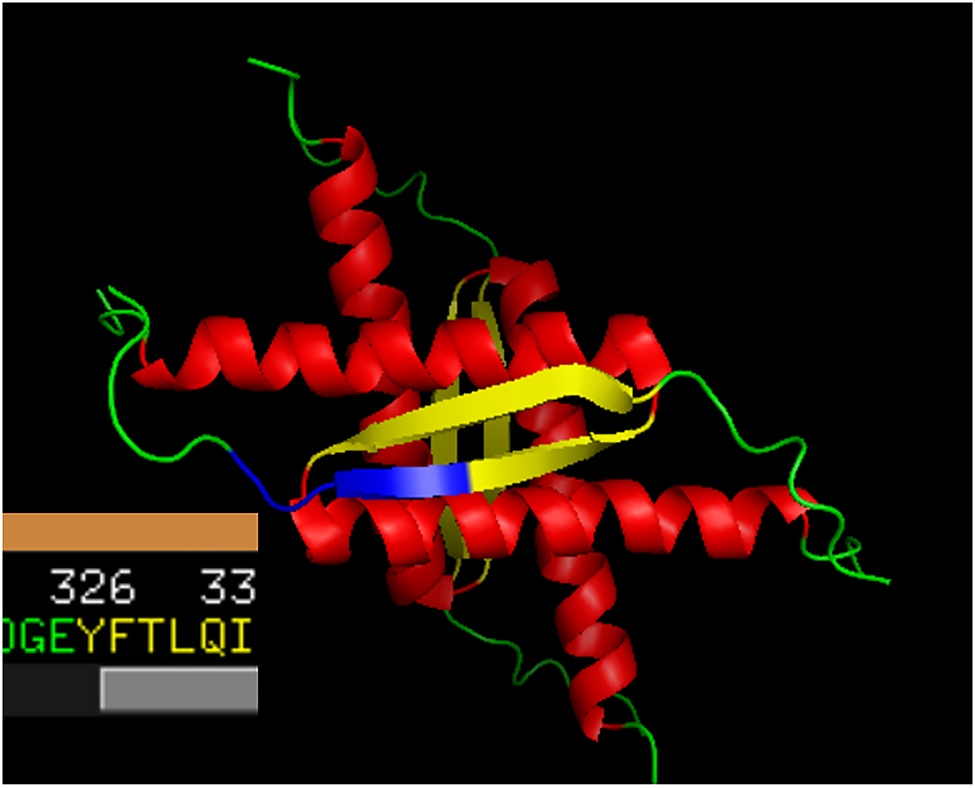

Supplement: Supplementary file 2 — Supplementary Material Details [file j_jib-2020-0043_suppl_002.jpg]

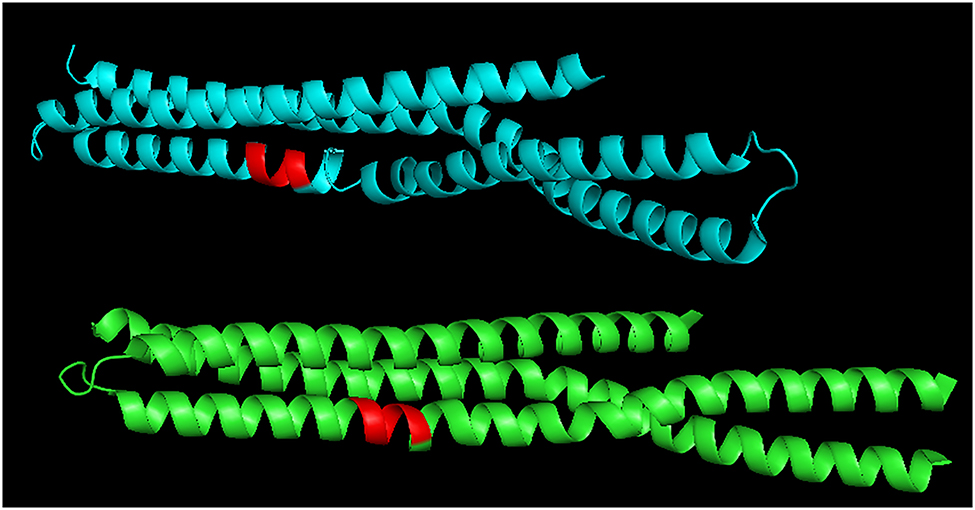

Supplement: Supplementary file 3 — Supplementary Material Details [file j_jib-2020-0043_suppl_003.jpg]

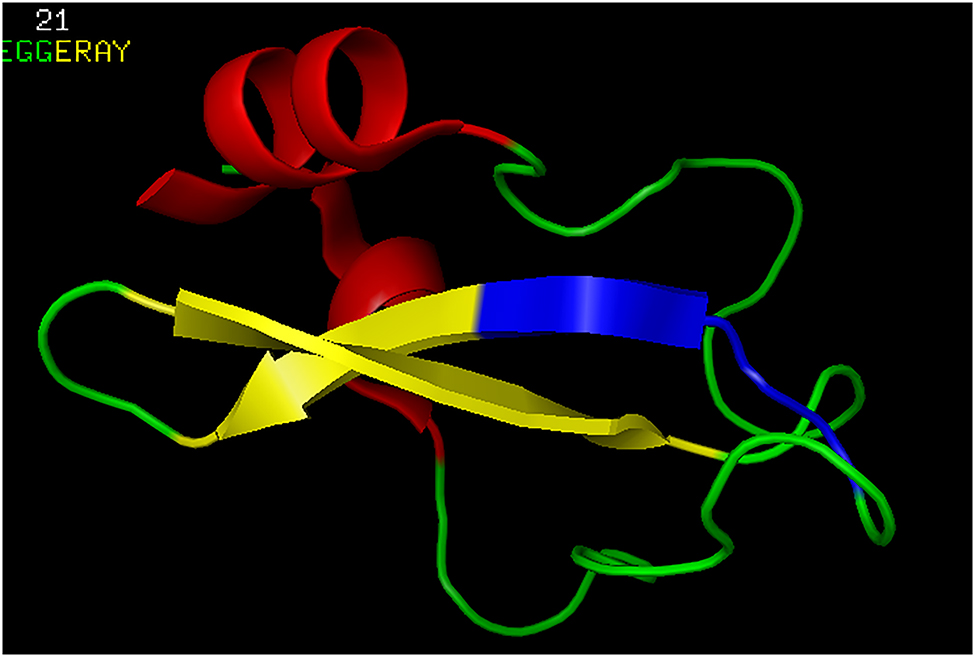

Supplement: Supplementary file 4 — Supplementary Material Details [file j_jib-2020-0043_suppl_004.jpg]
